# Daily Pleasure and Difficulties among Older Patients: Primary Care Setting in Japan

**DOI:** 10.31662/jmaj.2025-0002

**Published:** 2025-05-26

**Authors:** Junki Mizumoto, Hirohisa Fujikawa, Masashi Izumiya, Masato Eto

**Affiliations:** 1 Department of Medical Education Studies, International Research Center for Medical Education, Graduate School of Medicine, The University of Tokyo, Tokyo, Japan; 2 Center for General Medicine Education, School of Medicine, Keio University, Tokyo, Japan; 3 Department of Family Practice, Ehime Seikyo Hospital, Ehime, Japan

**Keywords:** activities of daily living, ambulatory care, geriatrics, long-term care, needs assessment

## Abstract

**Introduction:**

Older adults undergo physical and social changes that adversely affect their health. However, the prevalence and distribution of patients’ pleasure and difficulties remain poorly documented. Moreover, the acceptability and feasibility of self-administered questionnaires concerning their social conditions among older patients are still uncertain. Our study aimed to elucidate the distribution of daily pleasure and difficulties among older patients and assess the acceptability of these screenings.

**Methods:**

Descriptive epidemiology was conducted at a single center in Japan. From April 2023 to March 2024, self-reported questionnaires on activities of daily living (ADL), daily pleasure and difficulties, and the acceptability of these questions were distributed to all patients aged 80 years or older. The participants’ long-term care (LTC) certification level, a nationwide measure in Japan, was used to assess their ADL.

**Results:**

A total of 359 patients participated, with 192 (53.5%) not applying for LTC certification, 57 (15.9%) at Support Levels, and 101 (28.1%) at Care Need Levels. Among them, 242 (67.4%) reported daily pleasures, 56 (15.6%) experienced bathing difficulties, 73 (20.3%) encountered excretion problems, and 34 (9.4%) faced excessive financial burdens. These difficulties and financial burdens were more prevalent among those at Support Levels and Care Need Levels than those without LTC certifications. Most participants found these surveys acceptable.

**Conclusions:**

Participants requiring LTC reported daily pleasures as frequently as those without LTC certification. However, those requiring LTC more frequently reported difficulties in daily activities and faced excessive financial burdens. Healthcare professionals should inquire about older patients’ specific daily living needs.

## Introduction

Older adults are at risk not only of physical changes due to aging but also social changes such as retirement, exercise, social support, and nutrition, all of which affect health. These changes can lead to further deterioration in health status and physical function and poor social engagement ^(1)^. To provide personalized interventions to older patients, healthcare professionals must understand patients’ pleasures and difficulties in daily living ^(2)^. The proportion of patients with unmet social needs is generally high, and understanding patients’ pleasure and difficulties in daily living may be associated with the quality of geriatric care ^(3)^ and appropriate physician visit behavior ^(4)^. Although primary care has a strategic position in addressing patients’ psychosocial factors, the prevalence of these factors remains uncertain ^(5)^.

Most older patients require various forms of assistance in daily living. In Japan, individuals aged 65 years or older can apply for long-term care (LTC) certification if they currently require or are expected to require LTC. Based on their condition, applicants are classified as not applicable, in need of support, or need of care. There are 7 levels of LTC certification: Support Levels 1 and 2 (for those who need help with daily activities) and Care Need Levels 1 through 5 (for those who need help with daily activities such as eating and bathing). A higher level indicates a greater need for nursing care. Once approved for LTC, individuals are eligible to receive services based on their level of need ^(6)^. This classification is used nationwide in Japan and has been clinically validated ^(7)^. Typically, individuals at Care Need Level 2 have severe limitations in instrumental activities of daily living, while those at Care Need Level 3 have severe functional limitations in basic activities (e.g., ambulation, bathing) ^(8)^. Such LTC is necessary for patients to enjoy their lives, but few studies have examined how patients’ pleasure and difficulties in daily living are related to their LTC status.

Patient acceptability also has significant implications for implementing health-related interventions that address patients’ pleasure and difficulties in daily living ^(9)^. Previous research suggests that most patients consider screening for social risks to be appropriate ^(10)^. However, there is a dichotomy in patient perspectives: while some acknowledge the importance of screening for social risks, they may perceive the healthcare sector’s ability to address or mitigate these risks as limited, believing it to be outside the purview of healthcare professionals ^(11), (12)^. Preferences for screening procedures vary, with some advocating for brief and straightforward screening during outpatient waiting times ^(4), (13)^, while others express a preference for face-to-face discussions ^(14)^. Older patients tend to be more reluctant to undergo screening, and when they agree, they often prefer face-to-face interactions ^(15)^. The acceptability and ability of older patients to complete self-administered questionnaires about their social conditions remains uncertain.

This study aimed to elucidate the prevalence and distribution of pleasures and difficulties in daily living among older patients aged 80 years and older who regularly attend a primary care hospital and to assess their acceptability of a comprehensive self-administered screening for these factors.

## Materials and Methods

### Setting

This descriptive study took place at an outpatient department for general internal medicine and family medicine within a small hospital situated in a suburban region of Japan. This outpatient department serves as a primary care center, as most outpatient departments in small hospitals in Japan do ^(16)^. Other departments in the hospital include orthopedics, pediatrics, psychiatry, and surgery. With approximately 230 outpatient visits daily, the hospital caters to a population of approximately 500,000 individuals, including around 48,000 individuals aged over 80. Positioned along a national road, the hospital is surrounded by residential neighborhoods, with the nearest train station accessible via a 20-minute walk. There is no street bus stop in proximity, and a free shuttle bus operates between the station, residential areas, and the hospital.

### Questionnaire

To collect information on patients’ social backgrounds and their acceptance of such questions, the first author and the nursing staff in the department discussed and developed a self-administered questionnaire based on previous research ^(17), (18)^. The first questionnaire on patients’ social background (Supplemental File 1) included questions about the classification of certification for LTC (reflecting their activities of daily living), use of LTC services, daily pleasure and difficulties, and subjective economic burden. The rationale for collecting both LTC status and actual use of LTC services is to identify discrepancies between official LTC certification and real-world service utilization. Recognizing such discrepancies is crucial for healthcare professionals, as it allows them to assess whether patients are receiving adequate support and introduce necessary services accordingly. Although there are several validated assessments for activities of daily living, we focused on bathing and excretion to facilitate the response to the questions. The questionnaire also asked about support in daily life, and we are preparing another paper to report it. The second questionnaire was designed to assess the time required and patients’ acceptability about screening these factors (Supplemental File 2).

### Participants

From April 2023 to March 2024, we distributed the first and second questionnaires to all patients over 80 who had visited the outpatient department regularly. In Japan, the population aged 80 and above accounts for approximately 10% ^(19)^. This study focused on this age group to better reflect the realities of Japan’s aging society. Patients scheduled to be seen in their birth month were handed these questionnaires at the reception desk and asked to complete them while waiting for their consultation. Patients who were not scheduled in their birth month were given the questionnaires at the earliest visit after their birth month. Some patients could not complete the questionnaires because of physical and cognitive disabilities. In this case, their companions were asked to complete the form, or the nursing staff completed it based on the patient’s response.

### Analysis

We summarized the proportions of each response and evaluated the difference between participants at Support Levels, those at Care Need Levels, and those without any LTC certification using a chi-squared test via Microsoft Excel 2021. We deleted missing data.

### Ethical considerations

Patient consent was obtained on an opt-out basis. A notice about the study was posted in the clinic’s waiting room and on the website, providing patients who did not want their data used for the study with the opportunity to refuse. The study received approval from the Research Ethics Committee of the University of Tokyo Graduate School of Medicine and Faculty of Medicine (number 2022315NI). This study adhered to the Strengthening the Reporting of Observational Studies in Epidemiology Statement ^(20)^.

## Results

A total of 371 patients received questionnaires. Twelve patients declined participation, resulting in 359 participants. The median age of patients was 84 years (interquartile range [IQR]: 82-88). The number of female participants was 222 (61.8%) (Table 1).

**Table 1.** Participants’ Demographics (n=359).

Age [median (the first quartile-the third quartile)]	84 (82-88)
Gender, n (%) [woman/man]	222 (61.8%)/137 (38.2%)
Long-term care certification, n (%)	
Not applied	192 (53.5%)
Now applying	9 (2.5%)
Support Level 1	34 (9.5%)
Support Level 2	23 (6.4%)
Care Need Level 1	50 (13.9%)
Care Need Level 2	26 (7.2%)
Care Need Level 3	13 (3.6%)
Care Need Level 4	8 (2.2%)
Care Need Level 5	4 (1.1%)

Of the participants, 192 (53.5%) did not apply for LTC certification. The number of participants at Support Levels was 57 (15.9%), and at Care Need Levels, it was 101 (28.1%). There were few participants at Care Need Levels 3 or higher (Table 1). Among participants with LTC certificates, 18.4% did not use any services. The most utilized service was daycare service (rehabilitation daycare center or day health care center), used by 45.6% of applicable participants (Table 2).

**Table 2.** Long-Term Care Service Used by Applicants (n=158) (Multiple Answers Allowed).

Service	The number of users, n (%)
Not using	29 (18.4%)
Rehabilitation daycare center/day health care center	72 (45.6%)
Home-visit health care	28 (17.7%)
Short-term residential care	24 (15.2%)
Long-term residential care	22 (13.9%)
Home-visit nursing care	14 (8.9%)

### Daily pleasure and difficulties

The number of participants who reported having daily pleasures was 242 (67.4%). The numbers of participants who reported having trouble bathing, trouble with excretion, and excessive financial burdens were 56 (15.6%), 73 (20.3%), and 34 (9.4%), respectively (Table 3).

**Table 3.** Daily Pleasures and Difficulties (n=359).

	Yes	No	Not answering
Daily pleasure	242 (67.4%)	108 (30.0%)	9 (2.5%)
Trouble bathing	56 (15.6%)	302 (84.1%)	1 (0.3%)
Trouble with excretion	73 (20.3%)	283 (78.8%)	3 (0.8%)
Financial burden	34 (9.4%)	320 (89.1%)	5 (1.4%)

### Difference between LTC certification levels

**Figure 1.**
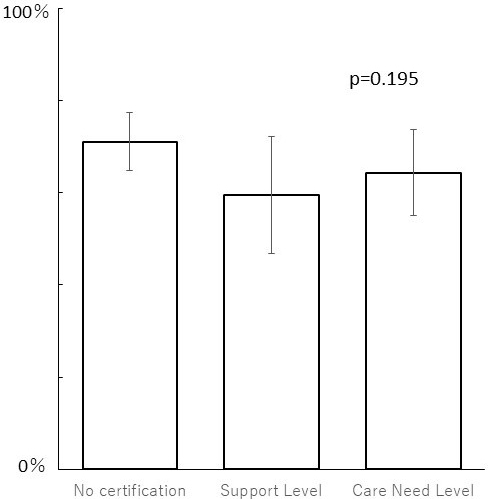
Daily pleasures among each long-term care status. A chi-square test showed no statistical significance.

The proportions of participants reporting daily pleasures did not statistically differ across LTC certification levels: without LTC certification (w/o): 71.1% (143/201); at Support Levels (S): 59.6% (34/57); and at Care Need Levels (C): 64.4% (65/101) (Figure 1).

**Figure 2.**
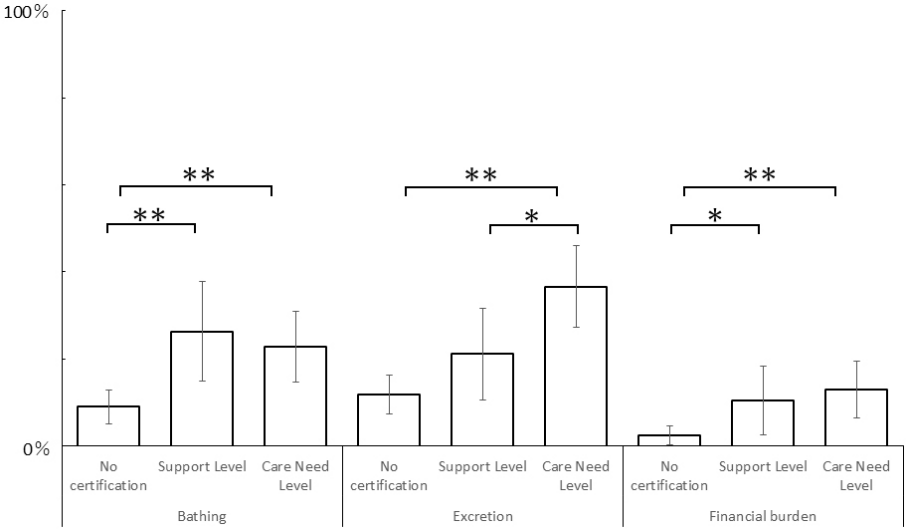
Difficulties in daily living among each long-term care status. Legends: *p < 0.05; **p < 0.005.

The proportions of participants reporting difficulties in bathing among w/o (9.0%: 18/201) were lower than S (26.3%: 15/57) and C (22.8%: 23/101). Those reporting difficulties in excretion among w/o (11.9%: 24/201) and S (21.1%: 12/57) were lower than C (36.6%: 37/101). Those reporting excessive financial burdens among w/o (2.5%: 5/201) were lower than S (10.5%: 6/57) and C (12.9%: 13/101) (Figure 2). Details of the responses to each question are shown in Supplemental File 3. Details of statistical analyses are shown in Supplemental File 4.

### Patients’ acceptability

The mean time to complete the questionnaire was 5.86 minutes (SD: 3.81). Of all 359 participants, 251 (69.9%) reported that it was easy to understand what to write down (Likert scale: 4 or 5), while 60 (16.7%) reported that it was difficult (Likert scale: 1 or 2). Additionally, 265 (73.8%) reported no discomfort in completing the questionnaire (Likert scale: 4 or 5), while 49 (13.6%) reported discomfort (Likert scale: 1 or 2). Furthermore, 204 (56.8%) agreed that this survey should be continued (Likert scale: 4 or 5), while 48 (13.4%) disagreed (Likert scale: 1 or 2) (Table 4).

**Table 4.** Patients’ Acceptability [n, %].

	Positive response	Neither	Negative response
Easy to understand what to write down	251 (69.9%)	48 (13.4%)	60 (16.7%)
Comfortable to complete the questionnaire	265 (73.8%)	45 (12.5%)	49 (13.6%)
Agreeable to continue this survey	204 (56.8%)	107 (29.8%)	48 (13.4%)

The median age of the 49 participants who reported discomfort with the survey was 84 years (IQR: 81-86). Among these participants, 30 (61.2%) were female, 30 (61.2%) were not certified for LTC, and 4 (8.2%) reported experiencing some financial burden. Additionally, 9 participants (18.4%) were at the Support Level, and 10 (20.4%) were at the Care Need Level. There was no statistically significant difference between participants who reported discomfort and those who did not (Supplemental File 5).

## Discussion

This study revealed several characteristics of older patients who regularly visit a primary care department in a suburban area in Japan. First, about half of the patients aged 80 years or older were not certified for LTC. This may be because advanced age does not always mean diminished activities (in Japan, a life expectancy is 84.5 years in 2021, and a healthy life expectancy―when a person can live without health problems limiting daily life―is 73.4 years) ^(21)^. Another possible explanation is that the need for LTC was not recognized. However, insufficient recognition may not be prevalent, considering that about one-fifth of participants had LTC certification but did not utilize any services. In Japan, the law mandates certification within 30 days of application for an LTC. However, the increasing number of applications has resulted in an average certification time of 36.2 days in 2021 ^(22)^. This extended timeframe may encourage patients to obtain certification before they need services pre-emptively.

Patients’ daily pleasures seemed maintained despite changes in their LTC status. LTC services may contribute to patients’ social participation and promote salutogenesis ^(23), (24)^. However, LTC services may fail to address patients’ basic needs adequately. In Japan, daily bathing in a bathtub is a cultural norm ^(25)^, and older individuals living in old houses often encounter obstacles such as steps to the bathroom and the depth and narrowness of bathtubs, which are not adequately addressed by the current LTC system ^(26)^. Research on caregivers assisting older adults with bathing is scarce. A 2001 report in Japan highlighted the problem that caregivers for bathing were often older spouses living together ^(27)^. This study suggests that the challenges associated with bathing care remain unresolved. Regarding excretion, older patients often resort to using portable toilets or diapers, as LTC services are seldom available at night. In older patients, bathing and toileting care needs are strongly associated with moderate dysfunction, and meeting these needs requires a variety of care, including home modification ^(28)^. The majority of family caregivers bear the physical, emotional, and financial burden of excretion care ^(29)^, and clinicians need to be aware that the basic needs of older patients, even if they are under LTC, may not be fulfilled.

About one-tenth of the participants reported an excessive financial burden. This proportion is lower than the relative poverty rate in Japan, which was approximately 16% in 2016 ^(30)^. One possible explanation is that regular visits to the hospital may help stabilize patients’ lives through social support. Another explanation is that the hospital may unintentionally exclude socially marginalized populations, or patients may hesitate to report their financial burdens. The economic burden is associated with various levels of reduced visits and worse patient experience ^(31), (32), (33)^, and failure to address these barriers in collecting patient-reported information may further increase health inequalities ^(34)^. Healthcare professionals should recognize these problems and be proactive in communicating with patients about their financial challenges ^(35)^.

The acceptability of this survey among patients was relatively high. A previous study reported that 65% of participants expressed comfort with documenting their social risks in the electronic health record, and factors influencing this receptiveness included patients’ previous experiences, trust in healthcare professionals, and being in a primary care setting ^(10)^. If patients perceive benefits from such surveys, their acceptability may be further enhanced. In this study, there was no statistically significant difference between participants who reported discomfort with the survey and those who did not. Unfortunately, the main factor seems to be insufficient power due to a small number of participants who reported discomfort, and further research should be warranted to confirm hypotheses related to this study (e.g., the relation between patients’ acceptability and their financial burden).

This study has some limitations. First, the survey was conducted in a single small hospital in a suburban area in Japan. Second, we use self-reported questionnaires, and the responses may not accurately reflect patients’ actual conditions. In addition, the questionnaires were not validated. While there are several validated assessments for activities of daily living, we focused only on bathing and excretion to facilitate participants’ response, which may not provide a comprehensive assessment of patients’ living conditions. Furthermore, the binary choice for daily pleasure may be overly simplistic, potentially overlooking nuances in patients’ experiences and well-being. Third, this study did not address how to approach patients’ difficulties, warranting further study. Previous research suggested that high utilizers who undergo screening for social needs rarely receive resources to meet those needs ^(36)^. In addition, there is limited research on staff acceptance of such screenings ^(37)^. Some clinicians may have concerns about the potential for increasing patient stigma ^(38), (39)^, highlighting the need to investigate the impact of screening on both patients and healthcare professionals.

In conclusion, this study examined the prevalence and distribution of both pleasures and challenges in daily living among patients aged 80 years and older who regularly visit a primary care hospital. The questionnaires took approximately 5 minutes to complete, and most of the participants did not feel discomfort in completing them. Professionals should recognize that older patients may have unique needs in daily living.

## Article Information

### Conflicts of Interest

None

### Author Contributions

Conceptualization, Methodology, Software, Formal analysis, Investigation, Data Curation, Writing - Original Draft, Visualization: Junki Mizumoto. Methodology, Validation, Formal analysis, Writing - Review & Editing: Hirohisa Fujikawa. Validation, Writing - Review & Editing, Supervision: Masashi Izumiya. Validation, Writing - Review & Editing, Supervision, Project administration: Masato Eto.

### Approval by Institutional Review Board (IRB)

The study received approval from the Research Ethics Committee of the University of Tokyo Graduate School of Medicine and Faculty of Medicine (number 2022315NI).

## Supplement

Supplemental Files
