# Supplementary material for: Daily Pleasure and Difficulties among Older Patients: Primary Care Setting in Japan
Source: JMA J. 2025 May 26;8(3):846–52. doi: 10.31662/jmaj.2025-0002 (PMC12328892; doi:10.31662/jmaj.2025-0002)
Supplement: Supplemental Files [file 2433-3298-8-3-0846-s001.pdf]

Supplemental File 1. The first questionnaire on patients' social background

**Basic information**	
**Name**	
**Age**	
**Gender**	
**Insurance and Service Availability**	
**Long-term Care Certificate Level**	
**Home-care Service Provider**	
**Care Manager**	
**Service**	Home-visit nursing:<br>Nursing station ( ) Frequency ( )[/week]
	Home help:<br>Care station ( ) Frequency ( )[/week]
	Home-visit rehabilitation:<br>Rehab station ( ) Frequency ( )[/week]
	Short-term stay at care facilities
	Other
**Contact**	
**Emergent Contact (First)**	Name:<br>Relationship:<br>Phone number:<br>Address:
**Emergent Contact (Second)**	Name:

	Relationship:<br>Phone number:<br>Address:
**Pleasure and difficulty**	
**Do you have something to look forward to in a daily life?**	Yes, No
**Do you have trouble in bathing?**	Independent, Partially dependent, Totally dependent
**Do you have trouble in excretion?**	Place: Home, Day-time care, Day-time rehabilitation, Home-visit bathing care, Other
**Do you have any economic burden?**	Yes, No
**Who usually does…**	
**Shopping**	
**Laundry**	
**Access to Hospital**	
**Any comment**	

Supplemental File 2. The second questionnaire on the time required and patients' willingness

**How long did it take you to complete the first questionnaire?**	[ ]min
**Did you find it easy to understand what to write down in the questionnaire?**	Definitely No (1) to Definitely Yes (5)
**Did you feel uncomfortable completing the questionnaire?**	Strongly Uncomfortable (1) to Not at all (5)
**Do you think it is beneficial for us to continue this survey?**	Definitely No (1) to Definitely Yes (5)

Supplemental File 3. Daily pleasures and difficulties at each LTC certification level

	All (n=359)	No certification (n=201)	Support level		Care Need Level				
			1 (n=34)	2 (n=23)	1 (n=50)	2 (n=26)	3 (n=13)	4 (n=8)	5 (n=4)
Daily pleasures	242 (67.4%)	243 (71.1%)	20 (58.9%)	14 (60.9%)	35 (70.0%)	16 (61.5%)	7 (53.8%)	5 (62.5%)	2 (50.0%)
Trouble bathing	56 (15.6%)	18 (9.0%)	10 (29.4%)	5 (21.7%)	11 (22.0%)	3 (11.5%)	7 (53.8%)	1 (12.5%)	1 (25.0%)
Trouble with excretion	73 (20.3%)	24 (11.9%)	7 (20.6%)	5 (21.7%)	19 (38.0%)	6 (23.1%)	9 (69.2%)	2 (25.0%)	1 (25.0%)
Excessive financial burden	34 (9.4%)	5 (2.5%)	3 (8.8%)	3 (13.0%)	9 (18.0%)	1 (3.8%)	2 (15.4%)	0 (0.0%)	1 (25.0%)

Supplemental File 4. Statistical analysis between each long-term care certification level

	No certification (n=201)	Support Level (n=57)	Care Need Level (n=101)	Chi-squared test (p-values)			
				3-item	No certification vs Support Level	No certification vs Care Need Level	Support Level vs Care Need Level
Daily pleasures	71.1% (143/201; 95% CI: 64.9%-77.4%)	59.6% (34/57; 95% CI: 46.9%-72.4%)	64.4% (65/101; 95% CI: 55.0%-73.7%)	0.195	NA	NA	NA
Trouble bathing	9.0% (18/201; 95% CI: 5.0%-12.9%)	26.3% (15/57; 95% CI: 14.9%-37.7%)	22.8% (23/101; 95% CI: 14.6%-31.0%)	<0.001	<0.001	0.001	0.002
Trouble with excretion	11.9% (24/201; 95% CI: 7.5%-16.4%)	21.1% (12/57; 95% CI: 10.5%-31.6%)	36.6% (37/101; 95% CI: 27.2%-46.0%)	<0.001	0.003	<0.001	<0.001
Excessive financial burden	2.5% (5/201; 95% CI: 0.3%-4.6%)	10.5% (6/57; 95% CI: 2.6%-18.5%)	12.9% (13/101; 95% CI: 6.3%-19.4%)	0.001	0.008	<0.001	0.006

Supplemental File 5. Statistical analysis between participants who were uncomfortable with the survey and those comfortable.

	**Discomfort (n=49)**	**No discomfort (n=310)**	**Chi-squared test (p-value)**
Age [median (the first quartile-the third quartile)]	84 (81-86)	84 (82–88)	NA
Gender, n (%) [woman/man]	30 (61.2%)/19 (38.8%)	192 (61.9%)/118 (38.1%)	NA
Not applied/Now applying LTC	30 (61.2%)	171 (55.2%)	0.48
Support Level	9 (18.4%)	48 (15.5%)	0.79
Care Need Level	10 (20.4%)	91 (29.4%)	0.09
Financial burden	4 (8.2%)	30 (9.7%)	0.53
